# Development and Validation of a Novel ELISA for the Specific Detection of Antibodies against *Mycobacterium avium* Subspecies *paratuberculosis* Based on a Chimeric Polyprotein

**DOI:** 10.1155/2021/7336848

**Published:** 2021-12-29

**Authors:** Roberto Damián Moyano, Magali Andrea Romero, María Alejandra Colombatti Olivieri, María Fiorella Alvarado Pinedo, Gabriel Eduardo Traveria, María Isabel Romano, María Natalia Alonso

**Affiliations:** ^1^Instituto de Biotecnología, Instituto Nacional de Tecnología Agropecuaria (INTA), Instituto de Agrobiotecnología y Biología Molecular (IABiMo), Consejo Nacional de Investigaciones Científicas y Técnicas (CONICET), Nicolás Repetto y Los Reseros, P.O. Box 1686, Hurlingham, Buenos Aires, Argentina; ^2^Consejo Nacional de Investigaciones Científicas y Técnicas (CONICET), Godoy Cruz 2290 (C1425FQB), CABA, Argentina; ^3^Centro de Diagnóstico e Investigaciones Veterinarias (CEDIVE) de la Facultad de Ciencias Veterinarias, Universidad de La Plata, Alvear 803, P.O. Box 7130, Chascomus, Buenos Aires, Argentina

## Abstract

Bovine paratuberculosis (PTB) is caused by *Mycobacterium avium* subsp. *paratuberculosis* (MAP). The optimization of detection tests specific for MAP is crucial to improve PTB control. In this work, we aimed to develop and validate a diagnostic tool based on an ELISA to specifically detect anti-MAP antibodies from bovine serum samples. For that purpose, we designed a recombinant polyprotein containing four specific antigens from MAP and optimized the ELISA. The validation consisted of the assessment of 10 sera from PTB-infected and healthy bovines with different OD values. The diagnostic performance of the polyprotein-ELISA was evaluated by testing 130 bovine serum samples (47 healthy, 48 MAP-infected, and 35 *M. bovis*-infected bovines). The ELISA using the polyprotein yielded an area under the ROC curve (AUC) of 0.9912 (95% CI, 0.9758–1.007; *P* < 0.0001). Moreover, for this ELISA, the cut-off selected from the ROC curve based on the point with a sensitivity of 95.56% (95% CI, 0.8485–0.9946) and specificity of 97.92 (95% CI, 0.8893–0.9995) was 0.3328. Similar results were obtained with an ELISA using the commercial Paratuberculosis Protoplasmatic Antigen (PPA). However, the ELISA with the polyprotein antigen showed a better performance against sera from animals infected with *Mycobacterium bovis* compared to the ELISA with PPA: lower cross-reactivity (2.85% versus 25.71%). These results demonstrate a very low cross-reactivity of the polyprotein with antibodies present in serum samples from animals infected with *M. bovis.* The designed polyprotein and the validated ELISA could be very useful for the specific identification of MAP-infected animals in herds.

## 1. Introduction

Bovine paratuberculosis (PTB) or Johne's disease, an endemic disease in many parts of the world, is a highly contagious chronic progressive granulomatous enteritis responsible for considerable losses to livestock and associated industries [1], whose etiologic agent is *Mycobacterium avium* subsp. *paratuberculosis* (MAP) [2]. The most common route of infection is by ingestion of contaminated milk, colostrum, or feces [3]. Calves up to 6 months of age are at higher risk of getting infected but the risk drops afterwards [4]. The entry of MAP is mediated by intestinal M cells and preferentially resides in phagosomes or early endosomes of host macrophages, predominately those associated with ileal Peyer's patches [5].

Clinical signs appear in advanced stages of the disease, which makes its diagnosis very difficult and, in turn, favors the spread of the pathogen in the herds [6]. Although the microorganism generally causes chronic granulomatous enteritis mainly in cattle, MAP can also affect other hosts such as goats, sheep, and deer [7–9].

Economic losses due to PTB are related to death, early elimination of animals, increased susceptibility to other infectious diseases, reduction of milk, meat, and reproductive yields, among others [10–12]. In addition, the association of MAP with Crohn's disease (CD) in humans, which is an autoimmune disease related to chronic intestinal depletion, suggests a zoonotic relevance [13].

Currently available commercial vaccines, which are based on inactivated strains, have been effective in decreasing both the elimination of mycobacteria through feces and the percentage of animals with clinical symptoms; however, they fail to prevent MAP infection [14, 15] and can interfere with the diagnosis of bovine tuberculosis (bTB) [16]. For this reason, herd-management programs, which consist in separating or eliminating PTB-infected animals [17], are an example of the main strategies used in several countries to control PTB [18]. The success of these PTB control programs, however, depends on the performance of the diagnostic tests used.

Control and eradication of PTB are difficult because of its long incubation period and the low sensitivity of the diagnostic tests to detect animals that are in early stages of the disease. The initial exposure to MAP leads to an important T-cell response characterized by release of proinflammatory cytokines such as gamma interferon (IFN-*γ*), interleukin-1 (IL-1), IL-6, and IL-2 [19]. Therefore, the measurement of secreted IFN-*γ* is a valuable tool for the detection of animals infected with MAP in early stages of the infection [20].

The specificity of the tests based on IFN-*γ* assessment, however, is low in animals below 16 months of age [21]. Moreover, the identification of IFN-*γ*-positive animals should be followed by other diagnostic methods such as ELISA or MAP fecal shedding to evaluate disease's progression in infected herds [22, 23]. Among the diagnostic tests, culture of MAP from feces, milk, blood, and tissues of infected animals is considered “gold standard” for the detection of MAP infection [24]. MAP isolation, however, is very expensive, laborious, and time-consuming and requires several decontamination steps during the process [25]. The sensitivity of fecal culture is ∼70% in clinically infected cattle but only 23–29% in subclinical PTB infected cattle. Conversely, real-time PCR (qPCR) assays have a higher sensitivity than the fecal culture and provide a rapid and specific PTB diagnosis. However, PCR-based tests are not a common practice in veterinary laboratories because of their cost [26].

Serological tests, particularly ELISAs, are of low cost, easy to perform, and readily automated for high sample throughput. ELISAs to detect antibodies against MAP can be applied for samples of sera or milk (for individual or bulk milk tank samples) [27]. The sensitivity of the ELISA test varies according to the stage of the disease (low in early and subclinical stages), level of MAP shedding in feces, and the age of animals [28]. Indeed, the ELISA detects about 30–40% of cattle identified as infected by culture of feces on solid media [29]. The improvement of the sensitivity and specificity of the serological tests requires the identification of well-defined antigens, even more considering that a single antigen hardly detects all the animals that are at different stages of the disease. The antigen candidates used in the available ELISAs so far include crude MAP cellular extracts, commercial Paratuberculosis Protoplasmatic Antigen (PPA), secreted antigens, cell wall and membrane antigens, lipoproteins, heat shock proteins (HSP), and recombinant proteins [30–35].

In this sense, the use of antigenic cocktails or polyproteins with specific epitopes can be an interesting alternative to increase the chances of detecting infected animals at different stages of the disease, mainly infected animals in subclinical stages of infection, when MAP shedding and immune responses are not so evident.

The aim of the present study was to develop and validate an ELISA for specific detection of antibodies against *M. avium* subsp. *paratuberculosis* in cattle sera. For this purpose, we have designed a chimeric polyprotein that contains the linear sequence of four epitopes of the reference strain of MAP K-10.

## 2. Materials and Methods

### 2.1. Chimeric Polyprotein Design and Protein *E. coli* Expression

*Escherichia coli* BL21 (DE3) pLysS was used for recombinant protein production, with the addition of ampicillin (100 *μ*g/mL) to the Luria Bertani (LB) broth or agar, when necessary.

The protein sequences of the four antigens (MAP0038, MAP0209c, MAP2513c, and MAP1589c) of MAP K-10 selected for the design of the polyprotein were obtained from Uniprot database (http://www.Uniprot.org). The B-cell epitopes from the antigen sequences were predicted using the BepiPred-2.0 program (http://www.cbs.dtu.dk/services/BepiPred/) [36]. The recombinant polyprotein, synthesized by Genscript (Jinsite Science and Technology, Nanjing, China), had 593 nucleotides and a fusion of four B epitopes with a six-His tag at the 3′ end of the sequence. The polyprotein was cloned in the vector pET-23a(+) and then expressed in *E. coli* BL21 pLysS by induction of 1.0-L LB broth cultures with 0.3 Mm isopropyl-*β*-d-thiogalactopyranoside (Sigma-Aldrich, Louis, MO) for 16 h at 20°C.

The resulting extracts were purified by chromatography affinity with a nickel resin, according to the manufacturer's instructions (Qiagen, Hilden, Germany). Elution fractions were pooled and dialyzed overnight in agitation in PBS at 4°C. Purified polyprotein aliquots were stored at −20°C. The degree of purification and concentration was evaluated on an SDS-PAGE gel with Coomassie Blue staining using a bovine serum albumin (BSA) concentration curve as the standard.

### 2.2. Polyprotein Expression and Antigenicity Evaluation

Proteins were fractionated on 12% SDS-PAGE and then stained with 0.25% Coomassie Brilliant Blue R250 (Sigma-Aldrich) or transferred onto nitrocellulose membranes (Hybond ECL, GE Healthcare). The expression of the polyprotein was assayed by Western blotting using a 1 : 3,000 dilution anti-His (GE Healthcare) as a primary antibody, and an alkaline phosphatase-conjugated anti-mouse IgG (Sigma-Aldrich) as a secondary antibody (1 : 3,000 dilution). A colorimetric detection was performed using BCIP/NBT (5-bromo-4-chloro-3-indolyl phosphate/nitroblue tetrazolium) Color Development (Promega), according to the manufacturer's instructions.

The polyprotein solution (100 *µ*L; 40 *µ*g/mL) was seeded and run in a denaturing 12% polyacrylamide gel with a single lane. Proteins were transferred to a nitrocellulose membrane (GE Healthcare), which was cut into strips of approximately 0.5 cm. Each strip was incubated for 1 h at room temperature with different bovine sera of known identity diluted to 1 : 100 in 5% nonfat dried milk/0.1% (v/v) in Tris-buffered saline 0.1% Tween 20 (T-TBS). Then, the strips were washed three times with T-TBS and finally incubated for 1 h at room temperature with a phosphatase-conjugated anti-bovine diluted to 1 : 5,000 in T-TBS as a secondary antibody. The colorimetric detection was performed using BCIP/NBT (5-bromo-4-chloro-3-indolyl phosphate/nitroblue tetrazolium), as previously described.

### 2.3. ELISA for Detection of Antibodies against MAP and *M. bovis*

Two different antigens for indirect enzyme-linked immunosorbent assays (ELISA) were used for the accurate detection of specific antibodies in sera of MAP-infected and healthy animals: PPA-ELISA and polyprotein-ELISA. The detection of antibodies of *M. bovis* in sera from infected and healthy animals was performed with a validated bTB ELISA that was previously used [37].

Briefly, polystyrene microtiter ELISA plates (Nunc MaxiSorp, Thermo Fisher Scientific, USA) were coated with 100 *µ*L of carbonate buffer (0.1 M sodium bicarbonate, 0.1 M sodium carbonate, pH 9.6) containing 4 *µ*g of either the polyprotein or the PPA antigen (Allied Monitor Inc., USA) or 33.8 ng of the antigenic mixture for the detection of *M. bovis* antibodies and subsequently incubated overnight at 4°C. The wells were then blocked with 0.2% porcine gelatin A (Sigma-Aldrich, USA) and then washed with PBST. Sera (100 *µ*L/well; 1: 100 dilution in PBS) were added and incubated for 1 h at 37°C. The wells were washed with PBST before adding peroxidase-labeled affinity purified protein G (BioRad Laboratories, USA) in a 1 : 4,000 dilution. Finally, the plates were washed, and the reaction was developed using hydrogen peroxide/2,2′-azino-bis(3-ethylbenzothiazoline-6-sulfonic acid) (ABTS, Sigma-Aldrich) in citrate buffer (pH 5), as the substrate/chromogen system.

For the evaluation of analytical sensitivity, a semiquantitative standard curve was performed using ammonium sulfate purified bovine immunoglobulin (0-1 mg) to correlate OD values versus micrograms/ml. Briefly, the purified bovine immunoglobulin was coated with 100 *µ*L of carbonate buffer for 1 h 37°C, subsequently blocked with 0.2% porcine gelatin A, and it was finally revealed using protein G-HRP and ABTS. The respective analytical sensitivity (detection limit) of the assay was determined using a 1 : 100 dilution of negative sera (*n* = 47). The analytical sensitivity was calculated using the formula [mean absorbance of the negative sera +2*σ* (standard deviation of the negative sera)] [38]. Also, twofold serial dilutions of five PTB-positive sera (1 : 100–1 : 6.400) were used for comparing both ELISAs.

The antibody reactivity of each sample was expressed with a corrected OD, OD_405_ (OD at 405 nm obtained in the sample wells minus OD at 405 nm in the control).

### 2.4. Serum Samples

The present study was carried out using 130 bovine serum samples from 47 healthy bovines (PTB/bTB-free), 48 MAP-infected bovines (PTB-infected), and 35 *M. bovis*-infected bovines (bTB-infected). The healthy animals belonged to PTB-free and bTB-free herds without signs of these diseases for more than 10 years and with negative results by PCR or culture from feces for MAP detection. A negative result for the tuberculin skin test (TST) and in the slaughter examinations for bTB detection was another condition to belong to the healthy group.

MAP infection in the 48 PTB-infected animals was confirmed by mycobacteria isolation from feces with further PCR amplification of the insertion sequence IS*900*. Finally, infection in the 35 bTB animals was confirmed according to necropsy performed in slaughterhouses authorized by SENASA. The tissues were tested by polymerase chain reaction (PCR), by amplifying the IS*6110*, which is an insertion sequence specific to organisms in the *Mycobacterium tuberculosis* complex [37]. These 35 animals belong to a herd that has been PTB-free for more than 15 years.

The Institutional Animal Care and Use Committee (CICUAE) of CICVyA-INTA, whose regulations agree with the European Union Laws for protection of experimental animals, authorized this study.

Aliquots of all sera were stored at −20°C until use.

### 2.5. ELISA Standardization and Repeatability

Different concentrations of the reagents and incubation times were assessed to optimize and standardize the test conditions. Subsequently, the repeatability was determined in the 10 serum samples of the analytical validation assay and expressed as the coefficient of variation of the corrected OD obtained in 30 runs in each of the samples used in the study. From the 10 serum samples used, 6 sera belonged to PTB-infected animals with a corrected optical density that ranged between 0.4 and 0.75 and 4 sera belonged to healthy animals (PTB/bTB free) with a corrected optical density <0.2. All sera were assayed in duplicate in every ELISA run.

### 2.6. Cut-Off, Sensitivity, and Specificity Determination by ROC Analysis

Receiver operator characteristic (ROC) curves of the PPA-ELISA and polyprotein-ELISA were performed using GraphPad software. For this purpose, the means of the corrected OD of the samples from animals classified as healthy (47 serum samples from PTB/bTB-free bovines) or from those confirmed to be infected by *IS*900 PCR amplification (48 serum samples from MAP-infected bovines) were used for this determination.

The optimal cut-off value for each ELISA was analyzed and determined by ROC analysis to obtain the best combination of sensitivity and specificity with 95% confidence interval (95% CI).

### 2.7. Data Analysis

Means, coefficients of variation, and standard deviations were calculated with Microsoft Excel for Windows. Statistical analyses were performed with GraphPad Prism 5.00 (GraphPad Software, USA). The means of the corrected OD obtained in the different groups were analyzed by Student's *t*-test. The analyzed groups were disease-free bovines (PTB/bTB-free), MAP-infected bovines (PTB-infected) or *M. bovis*-infected bovines (bTB-infected).

## 3. Results

### 3.1. Polyprotein Expression and Antigenicity Evaluation

**Figure 1:**
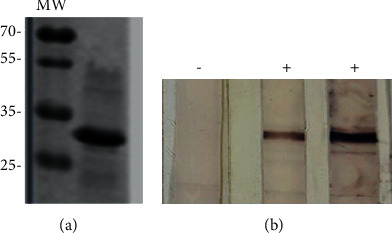
Polyprotein expression and antigenicity evaluation. Western blot using anti-His (1 : 3,000) as a primary antibody and an alkaline phosphatase-conjugated anti-mouse IgG as a secondary antibody (1 : 3,000). A colorimetric detection was performed using BCIP/NBT (5-bromo-4-chloro-3-indolyl phosphate/nitroblue tetrazolium) Color Development (Promega), according to the manufacturer's instructions (a). The antigenicity of the polyprotein was evaluated in a preparative 12% polyacrylamide gel with the polyprotein solution (100 *µ*l; 40 *µ*g/mL). The strips (∼0.5 cm) from a nitrocellulose membrane with the transferred proteins were exposed for 1 h at room temperature with different bovine sera (1 : 100) of known identity and after the corresponding washes were further incubated for 1 h at room temperature with a phosphatase-conjugated anti-bovine antibody (1 : 5,000), as a secondary antibody. The colorimetric detection was performed using BCIP/NBT as well (b). A representative image is shown. MW: molecular weight; −: negative serum sample; +: positive serum sample.

According to previous results of our group and other publications, the antigens selected for the design of the polyprotein of this study were those only detected in sera from animals infected with MAP but not with *M. bovis* [39, 40]. The recombinant polyprotein was successfully expressed, as evidenced by a Western blot using an anti-His antibody (Figure 1(a)).

The evaluation of the antigenicity of the obtained purified polyprotein consisted of adding and running 100 *µ*l of the solution in a denaturing 12% polyacrylamide preparative gel. Subsequently, the proteins were transferred to a nitrocellulose membrane and cut into strips, which were exposed to sera from healthy bovines or from bovines confirmed to be MAP-infected. An analysis of 15 sera from healthy (8 serum samples) or from MAP-infected (7 serum samples) animals revealed that only the sera from MAP-infected animals detected the polyprotein (Figure 1(b)). No unspecific signal was detected by sera from healthy animals.

### 3.2. ELISA Optimization and Analytical Validation

Firstly, the optimization of the ELISA technique consisted of analyzing different concentrations of the polyprotein as well as times of washing and incubations with ten sera from animals of various status and with different OD values (six positive and four negative). A sample containing only buffer was included as a negative control (buffer). The assay results indicated high repeatability, as evidenced by coefficients of variation below 25% (Table 1).

Regarding the evaluation of analytical sensitivity, both ELISAs detected the same limit of antibody titer in the tested sera, and the limit of detection was 1.49 *μ*g/mL for PPA-ELISA and 2.13 *μ*g/mL for polyprotein-ELISA.

### 3.3. Cut-Off, Sensitivity, and Specificity Determination by ROC Analysis

The diagnostic performance of the developed polyprotein-ELISA for MAP antibody detection was evaluated by testing 95 serum samples, which were classified as positive (48 serum samples from MAP-infected animals, PTB-infected) or negative (47 serum samples from healthy animals, PTB/bTB-free), according to their previous result in mycobacterium isolation in culture from feces with subsequent IS*900* PCR amplification. The ROC curve analysis was performed to both ELISAs to select the optimal cut-off values and to estimate the diagnostic sensitivities and specificities according to each possible cut-off point. Simultaneously, the same serum samples were analyzed in an ELISA that uses the commercial antigen PPA, which is routinely performed in the laboratory for the detection of antibodies against MAP.

**Figure 2:**
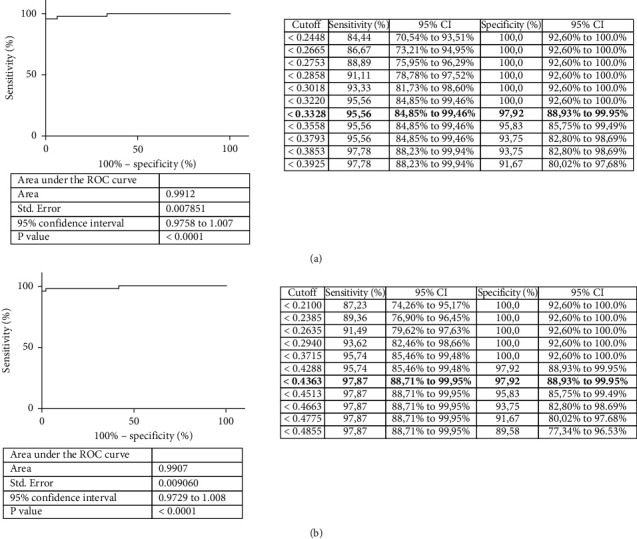
Diagnostic validation of polyprotein ELISA. (a) ROC curve analysis using the polyprotein (a) and PPA (b) as antigens and carried out with 95 sera samples, which were classified as positive or negative according to their previous result in mycobacterium isolation in culture from feces with subsequent IS*900* PCR amplification. The right panels display a list of the different possible cut-off points with their respective sensitivities and specificities. The chosen cut-off points with their corresponding sensitivity and specificity are indicated in bold.

The area under the ROC curve (AUC) was 0.9912 (95% CI, 0.9758–1.007; *P* < 0.0001) or 0.9907 (95% CI, 0.9729–1.008; *P* < 0.0001) for the ELISA using the polyprotein and that using the PPA antigen, respectively (Figure 2). The shape and the relevance of both AUC values demonstrated a high accuracy for both ELISAs.

The cut-off selected from the ROC curve for the polyprotein-ELISA based on the point with a sensitivity of 95.56% (95% CI, 0.8485–0.9946) and specificity of 97.92% (95% CI, 0.8893–0.9995) was 0.3328 (Figure 2(a)). Regarding the ROC curve for the PPA-ELISA, the cut-off selected was 0.4363, with a sensitivity of 97.87% (95% CI, 0.8871–0.995) and specificity of 97.92% (95% CI, 0.8893–0.9995) (Figure 2(b)).

**Figure 3:**
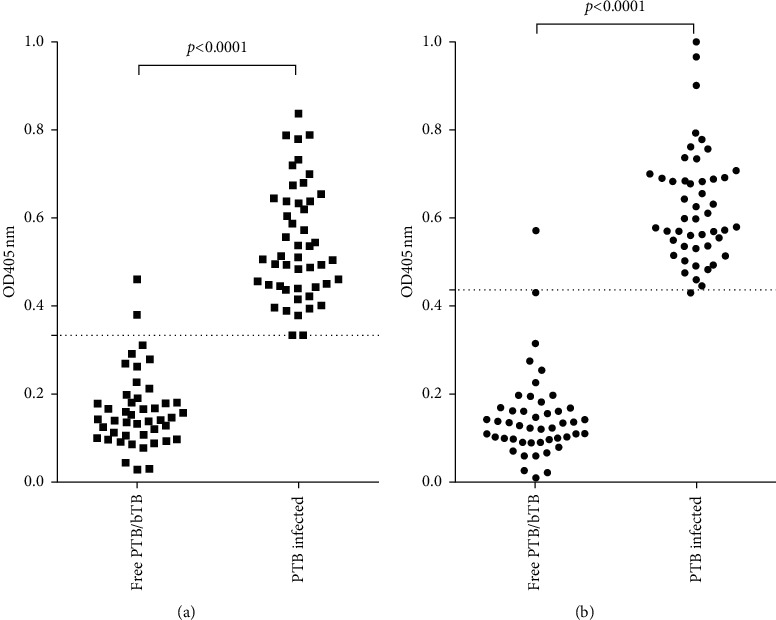
Evaluation of the ELISA developed using polyprotein as antigen and comparison with PPA-ELISA. Corrected OD at 405 nm (OD_405nm_ sample minus OD_405n_m buffer) obtained by the polyprotein-ELISA (a) and PPA-ELISA (b) of the 95 evaluated sera (47 sera from healthy bovines and 48 sera from MAP-infected bovines) for the diagnostic validation of the technique. The dashed line indicates the cut-off point selected by the analysis of ROC curves for each ELISA. Wilcoxon analysis showed significant differences between the PTB-free and PTB-infected groups (*P* < 0.0001).

According to the cut-off points selected for each ELISA, the evaluated antigen yielded two false positives for the healthy animals, whereas PPA only identified one false positive. On the other hand, no false negative reactions were observed in sera corresponding to MAP-infected animals with the polyprotein; conversely, the use of PPA as antigen yielded a false negative in one MAP-positive serum (Figure 3).

### 3.4. ELISA Cross-Reactivity in Bovine Sera Infected with *Mycobacterium bovis*

One of the main problems with the diagnostic methods used to detect MAP is the cross-reactivity with antigenic components present in other mycobacteria. For this reason, the following step was to evaluate the cross-reactivity with antigenic components present in *M. bovis* through the analysis of the optimized ELISA and the established cut-off point with sera from PTB/bTB-free healthy animals (PTB/bTB-free; *n* = 47) and *M. bovis*-infected animals (bTB-infected; *n* = 35).

**Figure 4:**
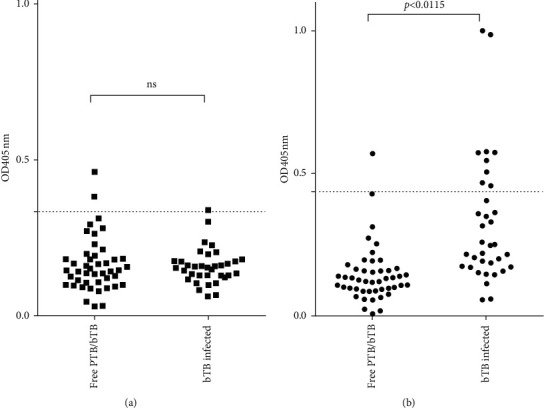
ELISA cross-reactivity in bovine sera infected with *Mycobacterium bovis.* Corrected OD at 405 nm (OD_405nm_ sample minus OD_405nm_ buffer) obtained by the polyprotein-ELISA (a) and PPA-ELISA (b) of the 95 evaluated sera (47 sera from healthy bovines and 35 sera from *M. bovis*-infected bovines) for the analysis of the cross-reactivity with *M. bovis*. The dashed line indicates the cutoff point selected by the analysis of ROC curves for each ELISA. Wilcoxon analysis showed significant differences between the PTB-free and TB-infected groups (*P* < 0.0001).

As a result, the ELISA using the polyprotein yielded as positive for MAP only one serum sample corresponding to animals infected with *M. bovis*, while the other ELISA detected nine false positives for MAP in sera from bTB-infected bovines (Figure 4). Thus, the polyprotein antigen performed better than PPA (cross-reactivity of 2.85% versus 25.71%, respectively).

## 4. Discussion

In recent years, important achievements have been made in the development of mycobacterial diagnostic methodologies. However, the optimization of detection strategies that specifically identify MAP-infected animals is still crucial. One of the reasons is that the specificity of the tests available so far is severely affected in the case of exposure or infections with other mycobacteria, because of the similarity of certain antigenic components between these organisms as well as for the slow growth, difficulty of isolation, among others [41–43]. The development and optimization of specific and sensitive MAP detection techniques allow more efficient control strategies that will have an important impact on reducing the prevalence of PTB in herds.

Currently, one of the serological tests most widely used for PTB detection is the indirect ELISA test that uses either the commercial antigen PPA or purified antigen extracts of MAP. Although several commercial serological tests (IDvet ELISA, IDEXX Laboratories ELISA, SERELISA® ParaTB Ab, among others) are currently available, they are expensive, use an additional preabsorption step with *M. phlei*, and display certain discrepancy in the ability to detect all infected animals [44, 45].

The specificity of ELISA varies between 40 and 100% depending on numerous factors, such as the antigen used, previous exposure to environmental mycobacteria, coinfection with other mycobacteria, and previous exposure to the tuberculin test for bTB detection [46]. In this sense, the choice of the antigen and/or antigens is crucial for the performance of the ELISA. Previously, researchers have used different biomarkers for the specific detection of MAP in sera from infected or exposed individuals, such as lipopeptides, L3P and L5P, PtpA and PknG secreted proteins, an Mce-truncated protein, and PPA-3 [32–35].

In the present study, we designed, expressed, and used a polyprotein as an antigen for the development of the ELISA, based on a previous evaluation of 54 proteins of MAP with sera from healthy or infected (with MAP or *M. bovis*) animals [39] and taking into consideration another previous study, where MAP1589c was specific for the diagnosis of PTB [40]. With all these data in mind, the selected antigens to design the polyprotein were MAP0038, MAP0209c, MAP2513c, and MAP1589c, since these antigens were only detected by the sera of MAP-infected animals, with no cross-reaction with sera from healthy animals or *M. bovis*-infected animals.

The polyprotein-ELISA developed showed a high accuracy (AUC .0.9912) with high sensitivity (95.56%) and specificity (97.92%) for the selected cut-off (0.3328). The experimental approach, however, presented certain limitations to evaluate the performance of the developed ELISA because of the complexity of the disease and the total number of animals analyzed.

The use of another widely used commercial antigen (PPA) allowed us to compare the performance of the developed ELISA. The results obtained for the evaluated parameters in both ELISAs were extremely similar. The main differences observed, however, were the results regarding the cross-reactivity in sera from animals infected with *M. bovis,* where the ELISA developed here showed a better performance.

Bovine paratuberculosis presents a wide spectrum of immunological and pathological stages associated with different phases of infection. In this sense, no single antigen could detect all infected animals [41]. This fact represents an important challenge in the selection of suitable antigens for the development of diagnostic techniques for PTB detection in herds. In this context, the use of antigenic mixtures or polyproteins may be an interesting alternative in the development of serological tests, since the presence of multiple epitopes increases the chances of detecting animals at different stages of infection. In a previous study, a MAP protein microarray performed with 868 purified recombinant proteins and evaluated with 180 bovine sera allowed the identification of several antigens that were recognized by MAP-infected bovine sera found at different stages of the disease. That research, however, lacks the analysis of cross-reactivity with other mycobacteria [30].

On the other hand, MAP infection increases the probability of an infection with *M. bovis* (2.35 times more probability of coinfection) and increases the susceptibility to other infections such as bovine mastitis [47]. Moreover, in the case of coinfected animals (animals infected with MAP and *M. bovis*), MAP infection interferes with the diagnosis of bTB, as evidenced by an increase of bTB false negatives with tuberculin skin test in bTB herds [47]. Roupie and coworkers have also detected a decrease in the sensitivity of an bTB-ELISA with antigen mixtures when a MAP and *M. bovis* coinfection takes place [48]. In fact, previous research has demonstrated that cattle naturally infected with *M. bovis* could produce false positive reactions when tested by various PTB-ELISAs [49].

These reports, among others, denote the importance of the specific diagnosis of PTB and bTB as well as of the assessment of both diseases in certain herds to avoid misdiagnosis. A misdiagnosis could induce to inadequate control measures of both diseases and subsequent economic losses because of the unnecessary elimination of false positive animals.

Based on all these reports, in this study, we decided to evaluate the performance of the ELISA developed in sera from animals infected with *M. bovis*. The cross-reactivity triggered with sera from *M. bovis*-infected animals using the selected polyprotein was very low, 2.85%, which shows that it can be very useful for the specific diagnosis of MAP.

## 5. Conclusion

The ELISA developed here could be a useful tool, since the polyprotein identifies MAP-infected animals, without displaying evident cross-reaction with animals infected with *M. bovis*. This will allow the application of more efficient sanitation strategies in herds with PTB alone or both diseases and this in turn would have an important benefit in controlling the spread of PTB. Furthermore, the use of the developed tool could be useful to assess the real prevalence of PTB in herds, carry out epidemiological studies of this disease, and monitor the progression of PTB in herds, among other things.

Although the results obtained so far are encouraging, more studies are necessary to deepen the diagnostic performance of the test which should be evaluated in prospective studies (including bTB-infected animals and other coinfections within the PTB group).

## Figures and Tables

**Table 1:** Results of repeatability assessment of ten sera, with mean, standard deviation (SD), and coefficient of variation (CV; CV = SD/mean) for each sample from 30 independent runs carried out on different days.

Sample	Mean	SD	CV
POS1	0.623	0.086	0.13804173
POS2	0.715	0.091	0.12727273
POS3	0.554	0.067	0.12093863
POS4	0.469	0.103	0.2196162
POS5	0.41	0.092	0.22439024
POS6	0.596	0.097	0.16275168
NEG1	0.112	0.009	0.08035714
NEG2	0.135	0.019	0.14074074
NEG3	0.109	0.007	0.06422018
NEG4	0.198	0.041	0.20707071
BUFFER	0.042	0.001	0.02380952

POS1–POS6 are positive samples with a corrected optical density that ranges between 0.4 and 0.75; NEG1–NEG4 are negative samples with a corrected optical density <0.2. BUFFER (PBS (Phosphate-Buffered Saline) alone) sample was used as control.

## Data Availability

The data used to support the findings of this study are included within the article. Also, extra information will be available from the corresponding author upon request.
